# Performing and Policing Prostitution: Race and Sexuality in Colonial Hong Kong Under the Contagious Diseases Ordinances

**DOI:** 10.1007/s00048-025-00417-5

**Published:** 2025-05-05

**Authors:** Ezra Kücken

**Affiliations:** Fachbereich 06 Geschichte und, Kulturwissenschaft, Philipps-Universität Marburg, Marburg, Germany

**Keywords:** Prostitution, Colonialism, Hong Kong, Sexually transmitted diseases, Intersectionality, Medical discourse, Prostitution, Kolonialismus, Hong Kong, Geschlechtskrankheiten, Intersektionalität, Medizinischer Diskurs

## Abstract

This paper delves into the complex construction and regulation of prostitution in colonial Hong Kong, exploring how intersecting dynamics of race, gender, class, and sexuality influenced perceptions and governance. Drawing on intersectionality, doing gender, and performativity frameworks, it analyzes the role of patriarchal imperialist capitalism, medical expertise, and humanitarian networks in shaping colonial attitudes toward prostitution, particularly through the enactment of contagious diseases (CD) ordinances. Despite existing scholarship on imperial regulation, there is a notable gap regarding the nuanced construct of the prostitute herself and its impact on regulatory practices. Through examination of historical documents, the paper reveals the interdependence between colonial and domestic constructions of race, sexuality, and class, highlighting their influence on legislative approaches in Britain and its colonies. By situating Hong Kong within broader imperial networks and scrutinizing medico-moral discourse and capitalist imperatives, the study exposes contradictions in colonial regulation, emphasizing the enduring legacies of exploitation. It advocates for further research into the performance and policing of prostitution, stressing the necessity of an intersectional lens to comprehend the complexities of colonial history.

## Abstract (de)

Der Artikel widmet sich der komplexen Konstruktion und Regulierung der Prostitution im kolonialen Hongkong und untersucht, wie sich überschneidende Dynamiken von *Race*, Geschlecht, Klasse und Sexualität auf Wahrnehmungen und Regulierungen auswirkten. Unter Anwendung der Theorien der Intersektionalität, *doing gender* und der Performativität wird die Rolle des patriarchalen, imperialistischen Kapitalismus, der medizinischer Expertise und der humanitären Netzwerke bei der Gestaltung kolonialer Paradigmen zur Prostitution analysiert, insbesondere hinsichtlich der *Contagious Diseases Ordinances* (CD). Trotz bestehender Forschung zur imperialen Regulierung existiert eine signifikante Lücke hinsichtlich der Konstruktion der Prostituierten selbst und ihrer Auswirkungen auf die Regulierungspraktiken. Durch die Untersuchung historischer Dokumente deckt der Aufsatz die Wechselbeziehungen zwischen kolonialen und domestischen Konstruktionen von *Race*, Sexualität und Klasse auf und hebt ihre Einflüsse auf gesetzgeberische Ansätze innerhalb Großbritanniens und seiner Kolonien hervor. Indem Hongkong in ein breiteres Netzwerk des Imperialismus einbettet und medizinisch-moralische Diskurse und kapitalistische Imperative genauer betrachtet werden, zeigt die Forschung Widersprüche in der kolonialen Regulierung auf und betont das anhaltende Erbe der Ausbeutung. Sie plädiert für weitere Erforschung zur Darstellung und Regulierung der Prostitution und betont die Notwendigkeit eines intersektionalen Ansatzes, um die Komplexitäten der kolonialen Geschichte zu begreifen.

“Time was when so-called Christian civilization seemed able to send its vices abroad and keep its virtues at home” (Andrew & Bushnell 1907: 13).

As the so-called “oldest profession” in the world, prostitution was widespread within colonial contexts. It comprised a locus of tension, contestation, and negotiation during a time marked by patriarchal, imperialistic capitalism and the rise of (medical) expertism and internationalism as well as empire-wide humanitarian networks. The contagious diseases (CD) ordinances serve as a multilayered illustration of how these intersecting forces shaped the colonial regulation of sex work. The methods and results of such imperial regulation have been studied by several historians. However, there is significantly less scholarship on the contested and multilayered construct of the prostitute herself and how this very construct influenced the configuration of the prostitute as a problem that required regulation.^1^

This article, then, investigates the construction and regulation of the prostitute in the context of colonial Hong Kong, which became the first colony within the British Empire to introduce CD ordinances in 1857, positioning it as a testing ground for policies that were later implemented in other colonial and metropolitan contexts. Drawing on the frameworks of intersectionality, doing gender, and performativity, it seeks to characterize the specific intersection of racial and sexual inferiority influenced by assumptions of gender and class inferiority that informed the construct of the colonial prostitute and imperial prostitution. Moreover, this article argues that the social and medical category of the prostitute constituted an intersectional identity necessitating performative acts, thereby rendering the performance of prostitution a crucial part of CD legislation and its successful application, as well as the rational for its particularity. Furthermore, the discourse surrounding the construction and performance of prostitution was connected to contemporary medico-moral debates, international humanitarian networks, and empire-wide dialogues negotiating slavery, agency, and modernity.

As Liat Kozma notes, “the life stories of the women in prostitution are often mediated through those who tried to reform, regulate or discipline them” (2017: 732). This observation applies equally to the sources available for the study of the CD ordinances in the British Empire, which primarily emanate from the bureaucracy, investigation, negotiation, and pathologizing that white Europeans imposed on racialized sex workers in British colonies. While these sources offer valuable insights into the British discourse and negotiation of these legislatures, it is imperative to be conscious of the linguistic and cultural barriers separating the colonizer and the colonized, as well as the immense bias and (attempted) homogenization of opinion employed by colonial and domestic administrators, physicians, military men, and humanitarians, whose accounts provide the basis for this work.^2^

Colonial and imperial sites constitute spaces of complex spatiality and temporality. As Philipp Howell asks, “When and where was the Empire?” (2017: 175) and Philippa Levine writes, “Empire and metropole were not separate sites; empire was not a single site” (2003: 4). It is crucial to consider this complexity when looking at the colonial legislation on prostitution and the CD ordinances in particular. Colonial actors as well as some modern scholars have sought to create a division between metropolis and periphery, justifying divergent forms of legislation in Britain and its colonies. However, Howell convincingly argues for “international legal and political regimes in which the British Empire was but one part” (2017: 176), a perspective employed in this paper to highlight the interconnectedness within the Empire and the CD ordinances.

Race and sexuality, alongside class and gender, represent interrelated categories in the history of the British Empire. The construction of these categories portrays the way in which concepts, ideas, and legislation were not exported fully formed to Britain’s colonies but were subject to the interdependence and interrelation of both domestic constructions and colonial experiences (Howell 2000: 321).

Following a brief summary and contextualization of the temporal and spatial characteristics that influenced CD legislation and application in Hong Kong, this paper focuses on the construction of prostitution and the figure of the prostitute through discussions of medico-moral discourse, the construction of the racial other, and the performance of white heteronormative masculinity and patriarchal imperialistic capitalism, drawing interim conclusions that inform the last chapter, which discusses the regulation of colonial prostitution through a focus on humanitarian networks and the inherent capitalism of the colonial enterprise. The conclusion includes possible focal points for further research.

## Contagious Diseases Ordinances in Colonial Hong Kong, 1857–1888

In 1841, Hong Kong became a British colonial possession and crown colony. As one of the four largest ports in the world with no other valuable natural resources and economic advantages, business and trading became the defining enterprises for the colony’s fiscal success and prestigious status.^3^ Its main population and the most integral part of its economy consisted of single male workers emigrating from China (Levine 1998: 680), leaving behind wives and families. These circumstances made them ideal for colonial capitalistic exploitation: without having to provide for a family and being accustomed to the local climate, they supplied the efficient but cheap labor needed for the colony’s economic and political stability and success. At the same time, this dominance of single male workers was perceived as a threat to colonial stability due to their inherent “foreignness” and the absence of their “families, in the proper sense of the word,”^4^ which necessitated the availability of sex workers^5^ and, following the logic of colonial administrators, justified the colonial regulation of the sex trade (Levine 1998: 680).

In his dispatch on August 27, 1856, Mr. Labouchere wrote:

“the Colonial Government has not, I think, attached sufficient weight to the very grave fact that in a British Colony large numbers of women should be held in practical slavery for purpose of prostitution, and allowed in some cases to perish miserably of disease in the prosecution of this employment, and for the gain of those to whom they suppose themselves to belong. A class of persons who by no choice of their own are subjected to such treatment have an urgent claim on the active protection of Government. I am not at present prepared to say, and I wish you seriously to consider, in what shape and to what extent it is practicable to give this protection. But I do not see how it can be given at all till the establishments in which such practices are supposed to exist brought under the eye, and in some measure under the control, of Government. On these ground, therefore, … I think that these houses of ill-fame and their inmates should be registered, and subjected to police regulations, in the first instance, of a sanitary character; that a strict medical inspection should be enforced, and that all diseased persons should be removed to hospitals and placed under treatment.”^6^

He thereby outlined the fundamentals of regulation of prostitution in colonial Hong Kong: registration of prostitutes and brothels, regular and compulsory medical examinations, sanitary detention in so-called lock hospitals of those found to be diseased, licensing and inspection of brothels, spatial segregation of so-called brothel districts from respectable parts of the city, persecution of unlicensed brothels and unregistered sex workers as well as police enforcement of regulation.^7^ These aspects were generally found in most colonial legislature on regulation of prostitution; however, they varied in practice and created what Howell calls a “heterogeneity of regulationism” (Howell 2000: 323). Despite its diverging application, all ordinances relied on the same assumptions about race and sexuality, as well as class and gender, outlined in this paper.

Colonial legislation of prostitution in Hong Kong predated the domestic contagious diseases acts first passed in 1864, negating the assumption that metropole concepts were exported to be employed in colonial contexts and instead highlighting an example of colonial spaces as places of experimentation and legislation.^8^ In Britain, CD acts were restricted to garrison towns with military and naval populations. They subjected sex workers to regular medical examinations and detention in lock hospitals in case of disease while legalizing the arrest and medical examination of alleged sex workers, who could then be sentenced to regular gynecological examinations and incarceration. The licensing and regulation of brothels, however, is notably absent from domestic legislation.^9^ The 1857 CD ordinances in Hong Kong, therefore, underscore the colony’s unique role as both an experimental site for imperial governance and a unique point of reference in the broader history of colonial regulationism.

As was typical under most CD ordinances, sex workers were subjected to weekly medical examinations and if found infected were confined to lock hospitals. These institutions were central to the colonial enforcement of the ordinances, serving as sites where medical treatment, disciplinary measures, and social control intersected. Designed primarily to treat venereal diseases, they disproportionately targeted women identified as prostitutes, subjecting them to compulsory examinations and detentions. These institutions embodied the punitive aspects of colonial health policies, often operating under conditions that more closely resembled prisons than hospitals, as the disciplinary regime within lock hospitals was characterized by strict rules and severe punishments, including solitary confinement and deprivation of food for perceived infractions. Physical conditions were frequently dire, with overcrowding, inadequate sanitation, and poorly maintained facilities exacerbating the suffering of detainees (Levine 2003: 70–74).

Ten years after its first passing, Ordinance 12 was replaced by Ordinance 10 of 1867 in its regulation of Hong Kong’s sex trade, focusing more on brothel keepers and others profiting from prostitution. It also placed the power of licensing brothels with the registrar-general and amplified police action under the inspectorate of brothels, providing both the registrar-general and the police superintendent with the right of entry without warrant to premises suspected to be unlicensed brothels. While under Ordinance 12 brothels were solely permitted in the Chinese district of Tai Ping Shan, Ordinance 10 altered the urban geography of sex work segregation by permitting the presence of brothels in more city districts, though they were still racially segregated.^10^ Brothels with European and American clientele were located in the central district and eastern part of the city, while Chinese–only brothels were confined to the western part,^11^ also home to the Tung Wah hospital, an important institution of the Chinese community that served as a lock hospital as well (Howell 2004: 235, 241 f.; Levine 1998: 677, 695).

Interestingly, Ordinance 10 facilitated a more thorough geographical regulation and segregation of prostitution than its predecessor, creating what Howell calls a “geography of racialized sexuality” (2017: 182), since more importance was placed on the segregation of brothel clienteles than on the segregation of the Chinese and European populations (Howell 2017: 182 f.; Howell 2004: 241 f.). Brothels were also segregated by class, dividing them in three classes based on their clientele and prices. According to the owner of a brothel that catered to captains and officers, charging premium rates, “my brothel is a first class one. No Europeans of inferior class go to my brothel; no soldiers or common sailors, and very rarely a Policeman.”^12^

This modus of regulation of prostitution is termed “regulationism,” a concept with contested origins as well as similarities and differences between metropole and colonial legislation and practice, aiming to maintain sexual and racial privileges—and, therefore, segregation—within colonial rule and white male superiority. Regulationism was informed by medico-moral discourse and practiced in various settings and forms throughout the empire,^13^ allegedly designed primarily for the combatting of venereal diseases (VD) (Howell 2017: 176, 191).

Regulationism reflected contemporary norms of class, gender, race, and sexuality, constructing the working-class woman of color as most deviant, culpable, and, therefore, in need of surveillance and (sexual) discipline. While white working-class women were rendered in a similar manner, colonial regulationism was more strict, thorough, and resistant to internal and external opposition and political pressure, augmenting the degree of culpability associated with colonial sex workers within CD ordinances.^14^ Colonial regulationism and its ensuing legislative initiatives were driven by multifaceted European apprehensions concerning race, sexuality, and the empire’s future trajectory. These concerns were intricately intertwined, with particular emphasis placed on anxieties surrounding interracial relationships and the specter of miscegenation (Howell 2017: 177 f.).

## Constructing Prostitution

Ordinance 12 of 1857 defines a prostitute as “any woman who shall live or reside in a registered or a declared brothel,” a rather broad and short–sighted definition insufficient for the combatting of “outdoor prostitution” or prostitution within unofficial and clandestine settings.^15^ To regulate prostitution and its agents, however, one needed to better discern who the prostitute was, not only according to her external circumstances but her very identity. For this, an essentializing of race, gender, and sexuality was necessary to construct the social and medical category of prostitute utilized both within legal and medical discourses.

Candace West and Don H. Zimmermann employ a thought-provoking concept when talking about the essentializing and biologicalization of gender: the trinity of sex, sex category, and gender. Sex (socially agreed upon biological traits such as genitalia or chromosomes, indicating a certain [binary] gender), sex category (socially recognizable physiognomic traits expressing a person’s “biological sex,” that is, specific hairstyles or gendered clothing), and gender (a person’s socially gendered behavior and activities, for example being soft-spoken, corresponding with their “biological sex”) are aspects that make up the category “gender” as an unchangeable, identity-constructing, and socially intelligible fact that every person possesses. The “correct” performance of that gender identity through gendered appearance and behavior is considered necessary and desirable in order for other people to “correctly” identify a person and, thus, have knowledge about that individual’s most basic characteristics, which, again, fall back on gendered ideas of being. Through socially agreed upon biological traits that are the first and most important indicators for a person’s gender, sex category and gender also become essentialized and biologicalized so that women are not only biologically in possession of a uterus, but also allegedly biologically weaker, more soft-spoken, and caring (West & Zimmermann 1987: 127).

This paper argues that the category of prostitute was similarly essentialized within CD ordinances. Through specific appearances and behaviors, women were categorized as prostitutes by police officers or informants and were thus subjected to medical examinations; findings of disease confirmed their identity as prostitutes. Sex workers could not simply be constructed as women performing work regarded as lowly and dirty, like other working-class informal labor, but needed to be seen as performing an identity that was framed as deviant and dangerous.^16^ However, which appearances and behaviors marked women as prostitutes? (Although not all sex workers were female, they remain the focus of both past and present research.) In other words, how did the CD ordinances and the discourse around them construct the figure of the prostitute?

## The Medical and the Moral

In 1869, the Hong Kong Colonial Surgeon, Dr. Murray, declared that the “sole object [of the CD ordinances was] restricting the extent of contagion and curing the disease.”^17^ In order for their containment and curing, however, the reason for the spreading of VD had to be rooted out.

STDs in the nineteenth century were subjected to intense moral and medical prejudice and regarded as directly linked to the dirty, deviant enterprise that was sex work. Prostitutes were quickly singled out as principal carriers of (then-called) VD, with their naming as such guaranteeing that the CD ordinances would become among “the principal arenas for the codification of female sexuality” (Levine 1998: 676) in Britain and its colonies. However, as Mary Poovey convincingly argues, prostitution was not discussed in the same manner as other issues related to female sexuality, such as pregnancy, but “transformed … into a social problem of such magnitude that by the 1840s this kind of female sexual behavior was being discussed in forms as various as newspapers, highbrow quarterlies, novels, and ‘scientific’ treatises on VD and sanitation” (1990: 30). This highlights the fact that while being female is part of a prostitute’s identity, prostitution itself is not a problem of women but a problem of prostitutes. It is an intersectional identity in its own right, albeit strongly informed by working-class and female codified presumptions.^18^

Definitions of prostitution and the bases for its construction were rather ambiguous and strongly impacted by medical, moral, and legal discourses. The nineteenth century still subscribed to the idea of female sexuality as venal, which informed the framing of prostitution as dangerous and deviant and its consequent need for definition, pathologizing, and regulation, as the modern state was responsible for the management of public health and morals through the managing of what Michel Foucault called “sexual discipline” (1980). Prostitution also embodied the intersection of class and gender prejudice (and, in the colonial context, racial prejudice): the lower classes of both Britain and its colonies were regarded as brute uncontrolled masses in contrast to the modern, restrained, and educated bourgeoise. Women and female sexuality were similarly seen to be in need of control and modesty, while male sexual urges were considered natural and thus deserving of legal recourse, so that, ironically, the very women providing such services were criminalized and pathologized. This is reflective of the Victorian double standard on sexuality and gender, which informed colonial understandings as well (Levine 1998: 675 f.; Howell 2000: 322).

The male-coded field of medicine, newly legitimated and authorized through the performance of expertism and internationalism, embodied a crucial role in the construction of the prostitute as a social and biological identity. CD ordinances were principally framed as sanitary measures related to what Levine calls the “newly fashionable idea of public health” (Levine 2001: 161), which can also be understood as a marker of modernity. The perspective on VD in these ordinances was heavily influenced by moral and religious views of sexual behavior, now seemingly modernized within the use of new scientific vocabulary, centering the question of culpability that was to be found in the social as much as in the individual body (ibid.).

Especially in the colonial context, however, the medical intent of the regulation of sex work seems to be rather secondary. Here, medicine appeared to perpetuate an image of a modern nation negotiating its anxieties about modernity in a space considered backward and very unmodern. While growing imperial influence and its accompanying status and prestige, as well as its recognition of medicine as a modern, allegedly rational understanding of the body, can be described as markers (and makers) of colonial modernity, the linking of science and sexuality is just as much a symbol of this very modernity. Within colonial legislation on VD, sexual activity was directly connected to the transmission of disease, therefore legitimizing a regulation of the former for the containment of the spreading of the latter. Additionally, medical and moral discourses were also intrinsically linked insofar as medical opinion was informed by contemporary assumptions and anxieties about promiscuity, while moral opinion appropriated more technical and scientific terms related to medical expertise to voice its preoccupations (Levine 2001: 160, 168; Levine 1998: 678).

Nevertheless, Britain’s principal concern was related to the potential weakening of the imperial military because of VD, which meant anxieties about prostitutes as carriers of VD, threatening Britain’s ability to maintain and expand its empire. This was seen as sufficient justification for both domestic and colonial regulation of prostitution, and the data confirms this.^19^ It also linked the military with medical experts: military doctors were at the forefront of new medical inventions; committees for CD ordinances consisted mostly of medical and military men with experiences in British colonies (with Malta frequently constructed as an example of a CD ordinance success story); and many advocates of CD ordinances had military ties and focused on soldiers’ health. The connection between the military and the colonial enterprise—and public health and modernity—was thus cemented in the discourse around VD and the construction of the figure of the prostitute (Levine 2001: 167–16.; Kozmat 2017: 741; Howell 2000: 326).

This relation also becomes apparent in the categorization of risk. Military men were seen as being “at-risk” and consequently in need of protection, while sex workers were identified as the risk that needed to be regulated (Levine 2001: 162). Interestingly, this made the female prostitute the active agent and reduced the male soldier to a passive receptor, which might have been the reason for the degree of emotionality accompanying discussions of VD: stereotypical gender roles of men as protectors and women as those in need of protection were reversed—even more so in the colonial context, where white men, the epitomes of strength and superiority, needed to be protected from women of color, contradicting their gender and racial identity based on masculinity and whiteness performed through vigor and dominance. Following this logic, female sex workers might also be constructed as committing a “double crime”: infecting innocent people with diseases while failing to perform their normative roles as women.

The regulation of prostitution within a medical-military framework was also clearly an expression of anxiety about miscegenation and interracial relationships, which could simultaneously be surveilled under the CD ordinances to a degree that “even when it was clear that the medical regulation of prostitution was not effective, regulatory measures were maintained because they served a secondary goal of maintaining order and supervising the conduct of both prostitutes and soldiers” (Kozma 2017: 741). This was also informed by an ideology of whiteness: soldiers were an imperial necessity, and so were their sexual behavior and marital status. Returning from service abroad, they were expected to seamlessly fall back into the heteronormative matrix of Victorian society, producing healthy citizens as their new eugenic duty to the nation. In order to do so, these soldiers needed to be free from diseases impacting future generations of Britain. As Levine candidly puts it, VD became a “deeply gendered metaphor for the health of the race and of the nation” (2001: 167).

The East was viewed in an Orientalist manner as a “sensual” place of squalor and backwardness where behavior and custom facilitated disease and vice-versa, in other words: the perceived chaos of indigenous culture and ways of living confirmed their complicity in the rapid spreading of diseases.^20^ As will be discussed later, this argumentation was also necessary for justifying the racial inequalities in access to the benefits of modernization, as it constructed the dichotomy of the unsanitary, promiscuous, “sensual” Chinese and their sexually disciplined, hygienic Western counterparts. All the while, this classification was portrayed as a result of allegedly neutral, unbiased scientific rationalism that masked racist colonial prejudices as empirical observations that could then be employed to further define the risk population that needed to be put under surveillance and upon whose correct identification the success of the CD ordinances relied (Levine 2001: 161). The face of this risk group was female, poor, and colonized, and its study, therefore, necessitates an intersectional lens.

This racial layer of the CD ordinances was replicated in the spatial and quotidian segregation within medical reports characterizing Asian living conditions as dirty and unhygienic—hygiene, in general, can be understood as an important marker of modernity and Britain’s alleged superiority—and thus the ideal basis for breeding and spreading diseases while simultaneously confirming their pre-modernness and subsequent inferiority. In an 1874 report, a Hong Kong Colonial Surgeon wrote, “without exception, these places [brothels] were filthy overcrowded dens … black with filth and smoke”; and in an 1879 report, “I’ve a pretty good stomach and don’t stick at trifles, but I found the inspection of these places [brothels] acted as a very unpleasant emetic.”^21^ As sex workers were already associated with filth and uncleanliness, Chinese sex workers were considered additionally unsanitary. Within colonial epistemology, this state of squalor and lack of hygiene was attributed to an individual failing due to racial inferiority instead of to the very living conditions produced by colonial capitalism and its ruthless striving for progress and profit. As already mentioned, colonialism, hygiene, and morality inhabited a highly charged field where cleanliness was required to be “civilized” (Levine 2001: 164). Following the hypothesis of female prostitutes as committing a “double crime,” Victorian gender roles and the close link between hygiene and femininity (and whiteness) highlight the way that racialized female sex workers were also incriminated by this shortcoming, which clearly illustrates the interconnectedness of race, sexuality, hygiene, and femininity. The colonial prostitute was thus constructed as a sexually deviant woman living in unsanitary conditions, bearing the markings of the intersection of class, gender, and racial inferiority.

## The Racial Other

While prostitutes both at home and in the colonies were regulated under CD ordinances, these varied widely in legislation and practice, necessitating a differentiation between colonial and domestic prostitutes as well as colonial and domestic prostitution within the construction of these categories. Crucial to this divergence between colonial and domestic regulationism was the pathologizing of Chinese as a racial and cultural other as well as the framing of the Chinese population as both the actual constructors of the system of regulationism and the reason for its application.

For this purpose, the indigenous Chinese population was collectively constructed as dangerous and pathological. This racial aspect of CD ordinances most strikingly appears in its actual enforcement: while all brothels and sex workers needed to be registered, only those with a US-European clientele were subject to regular, compulsory medical examinations. The implication of this can be most candidly summed up in the words of Registrar General Alfred Lister, “We were not trying to save the Chinese from syphilis” despite the manifold declarations of humanitarianism and accordance with the civilizing mission of the West discussed later in this paper.^22^ Nevertheless, the active campaigning, organizing, and disdain of the Chinese community regarding the examination of even this demographic of Chinese women was conceived as proof of their “natural” mistrust of “modern” Western medicine, bureaucracy, and state discipline and thus their unreadiness for Western modernity. This became the basis for what Howell terms the “discourse of difference” (2000: 181 f.), informing the particularities with which the CD legislatures were practiced. In other words:

“It was a wise recognition of the natural laws at work in this mass of corruption called prostitution, when the Government of Hongkong confined the application of the principal provisions of the Contagious Diseases Ordinance, viz., compulsory medical examination, to the licensed prostitutes in houses for foreigners only, and exempted from the same law the great mass of prostitutes for Chinese.”^23^

Another important aspect of this construction of racial otherness was the claim that “the system of prostitution which obtains in Hongkong is essentially Chinese and radically un-English.”^24^ Within colonial epistemology, Chinese prostitution was “essentially a bargain for money and based on a national system of female slavery, polygamy, and mui tsai.”^25^ This was a direct result of the guarantee made to the Chinese population after the British acquisition of Hong Kong in 1841 that they would be governed in accordance with their laws and customs, including practices regarded as backward and brutal, such as concubinage and the trafficking of girls and women.^26^ Regulationism was thus framed as specific to Chinese circumstances and therefore needed to be accepted by colonial authorities as normal and inherently Chinese.^27^ As one commissioner stated in 1879:

“Whatever vices the Chinese of Hongkong may have learned from foreigners, whatever evils the intercourse between foreigner and Chinese may have created, prostitution, brothels and the system of licensing brothels with a view to raise a revenue, legally or illegally, were indigenous institutions in China centuries before the present nations of Europe emerged from barbarism. When the Colony of Hongkong was first established … it was forthwith invaded by brothel keepers and prostitutes from the adjoining districts of the mainland of China, who brought with them the national Chinese system of prostitution, and have ever since laboured to carry it into effect in all its details.”^28^

Rather than addressing the colonial role in the organization and promotion of prostitution and the spreading of VD within colonizing and colonized populations, colonial epistemology constructed “Chinese social evil,” portraying the Chinese prostitute as an inevitable evil. Regulationism thus appears both as essentially Chinese and necessitated by Chinese conditions, with colonial pragmatism tolerating it despite its origin in a backward and brutal culture. This revisionist narrative, with its special focus on fiscal benefits derived from regulationism, considered that Chinese regulationism “originated and developed the practice of prostitution as a masterpiece of political economy, making it a source of revenue to the country”^29^ and was employed by both advocates and opponents of the CD ordinances (Howell 2004: 236 f.; Levine 1998: 691). Meanwhile, neither the Chinese population nor the prostitutes themselves were ever actually asked for their opinion^30^ (Andrew & Bushnell 1907: 37).

Another aspect of this paper’s intersectional lens is the interconnectedness of the British Empire in its construction and regulation of sex workers. The tools for gynecological examinations used on Chinese prostitutes were first utilized on enslaved Black women, and those who advocated for the repeal of CD ordinances—like Josephine Butler or Governor John Pope-Hennessy—built on the strategies of the anti-slavery movement to further their cause. Additionally, the empire-wide discourse on “white slavery” also depicts the interconnectedness of the racial layer of prostitution (Howell 2000: 336). Moreover, as Howell and Stoler argue, the white British middle class depended on “conflicts and comparison with this external racial other, as well as the internal sexual and class other” (ibid.) for the confirmation and construction of their own self-controlled, superior identity as members of the metropolitan bourgeoise (Stoler 1996: 193).

## Prostitution and (Colonial) Masculinity

The object of colonial (erotic) fantasies and curiosities, colonized women were alternately framed as exotic and hypersexual or oppressed and in need of saving by benevolent Europeans. At the same time, interracial sexual relationships were regarded as necessitating state interventions to uphold the prowess and stability of the empire. The brilliant postcolonial theorist Frantz Fanon wrote about the ways that colonial relations produced and channeled desire (Fanon 2004: 5): the colonial subject was seen as inferior and objectified and, as is apparent in much source material and research, female sexual desire was largely absent from the discourse around colonial sex work, while masculinity was the ever-present determinant force of such relations.

In the colonial context, it was not only about performing masculinity but performing white masculinity, hence confirming both sexual and racial dominance and superiority. Colonial authorities feared the emasculation of British men during their service in Britain’s Asian colonies for Orientalist colonial epistemology regarded Asian men as emasculated and weak (Levine 2003: 260, 263 f.; Sinha 1995). VD again served as an expression of this anxiety by embodying the threat of weakening soldiers and, by extension, the empire itself through interracial sexual contact regarded as unsanitary and dangerous. Furthermore, since Chinese sex workers were constructed as the principal carriers of VD in Hong Kong, they came to embody this threat of emasculation.

Again, this weakening and emasculation was seen as particularly problematic and threatening for soldiers returning home from duty and tasked with participating in procreation. According to the surgeon mayor of Netley Hospital, “[the returning soldiers were] utterly broken down in health, hardly able to crawl, covered with scabs and sores, with the foul odour of the disease about them, objects of disgust and loathing to themselves and all around them.”^31^ This expresses the particular danger associated with VD contracted in the so-called tropics: not only the transformation of strong, healthy Brits into unmanly and weak Eastern men, but the consequences this entailed for Britain’s future generations, both informed and consolidated by the ever-present threat of interracial intimate relationships.

Interestingly, while prostitution was equated with the transmission of VD and the subsequent weakening of Britain’s military prowess—which can be interpreted as a weakening of Britain’s white masculine superiority—prostitution was closely linked to the performance of masculinity and dominance, especially within the colonial context. Within this framing of nonwhite sex workers as active agents infecting innocent, passive white men with a vile disease—a reversal of gender stereotypes—colonial sex work needed to be regulated in order to reinstate white men’s control and dominance over colonized female bodies. Control over women’s bodies is always justified by men’s so-called natural urges and their supposed right to an outlet. Nevertheless, as Carole Pateman notes, “whether or not any man is able and willing to find release in other ways, he can exhibit his masculinity by contracting for use of a woman’s body”; therefore, prostitution is made necessary through the performance of masculinity, as “the exemplary display of masculinity is to engage in ‘the sex act’” (Pateman 1988: 199). Given the intersection of race and sexuality, this was especially crucial in the colonial setting, for not only masculinity but specifically white masculinity needed to be displayed.

Within colonial contexts, however, so-called sex acts needed to be regulated to secure the safety of performing this very masculinity since intimate relationships with colonial women in an unregulated setting signified the threat of emasculation, Orientalization, and the weakening of British men and soldiers. Regulationism, therefore, guaranteed white men’s secure and controlled access to colonized female bodies, enabling them to safely display their masculinity and superiority. At the same time, regulationism, according to Pateman, played a significant role in constructing sex work as a profession and identity, professionalizing the trade through the attachment of an intricate bureaucracy and sexual discipline in the form of surveillance and examinations and, most importantly, legitimizing prostitution through compulsory registration and licensing (Pateman 1988: 196 f.). This presents an important change brought about by the CD ordinances, as sex workers prior to regulationism were usually poor working-class women engaged in different kinds of informal labor, with sex work being only one among various jobs needed to make ends meet. These women were part of a diverse demographic reduced to the label “common prostitute” within both domestic and colonial CD ordinances. As both a profession and an identity, the figure of the prostitute needed to be homogenized in order to be intelligible and recognizable for others, especially the law enforcement tasked with identifying them. This, again, strengthens the argument that, through CD ordinances, the prostitute needed to be constructed as a social category informed by a biological reality interpreted by socially agreed-upon norms of race and sexuality, as well as class and gender.

Establishing a legitimate, secure, and accessible outlet for men’s urges in colonial contexts was even more necessary given the threat of satisfying them through other means, such as masturbation or homosexuality^32^ (Kozma 2017: 743). Thus, the regulation of prostitution was also justified as an accomplice in sustaining heterosexuality among European men in the colonies. As these men passed a significant amount of time away from their wives, they tended to form homosocial, sometimes homosexual relationships with their fellow soldiers (Potter 2015: 77; Levine 2003: 265, 292 f.), which made prostitution even more of a necessity as an outlet for their sexual needs that aligned with European expectations of heterosexuality.

In accordance with the double standard on sexual morality all too apparent in Victorian society, sex workers were not regarded as similarly engaging in natural urges and confirmation of heterosexual expectations. Regarding the domestic CD acts, the Royal Commission on the Administration and Operation of the Contagious Diseases Acts states that “with the one sex the offense is committed as a matter of gain; with the other, it is an irregular indulgence of a natural impulse,” virtually absolving men from their participation in prostitution while simultaneously reproaching the sex workers themselves.^33^ In the context of colonial Hong Kong, this double standard is deepened by the intersection of race and sexuality and echoes the statement of the Royal Commission in their argument that

“The principal points of difference between the various classes of Chinese prostitutes of Hong Kong and the prostitutes of Europe amount therefore to this, that Chinese prostitution is essentially a bargain for money and based on a national system of female slavery: whilst European prostitution is more or less a matter of passion, based on the national respect for liberty of the subject; and further that these Chinese prostitutes of Hong Kong are, as a whole, an extremely quiet, orderly, and well-behaved sort of people, not given to intemperance or excess; in one word, that they are not ‘abandoned women’ as the prostitutes of Europe too frequently are.”^34^

Subsequently, within colonial epistemology, the colonial sex worker was condemned for providing sexual services due to the alleged non-natural nature of female sexual urges and desires, while her white male customer was entitled access to her body due to his natural sexual needs vital to his performance of white colonial masculinity. At the same time, her engagement in prostitution due to her belonging to a cultural and racial other for whom this was an established institution was presented as inevitable and thus normalized. As Pateman poses in her book, “Why do men demand that satisfaction of a natural appetite must take the form of public access to women’s bodies in the capitalist market in exchange for money?” (1988: 198).

## Prostitution and Capitalism

The Chinese feminist He-Yin Zhen has written:

“Eating is the most important [thing in the world]. You who are women: what is it that makes one suffer mistreatment? It is relying on others in order to eat. Let us look at the most pitiable of women … Every day they [prostitutes] are beaten by their pimps. Whatever the customer is like, they must service him if he wants to be serviced … What is the reason for this? It is simply that since your family is poor you must sell yourself in this way in order to eat” Zhen 2000: 390).

By identifying sex workers as just one of many groups of laboring women suffering under patriarchal capitalism, she expands Karl Marx’s reference of prostitution as “a *specific *expression of the general prostitution of the *laborer*” (Marx 1964: 133 footnote) that fails to recognize the important nuance of the intersection of class and gender central to Zhen’s statement. The colonized working-class woman is, therefore, compelled to utilize the capital that is her body in order to survive under the oppressive systems of patriarchy, capitalism, and imperialism, merging together to form an intersectional form of subjugation. She is not free because, as Zhen states, she needs to eat and thus participates in these very systems.

Pateman defines freedom as “the unconstrained capacity of an owner (rational entity), externally related to property in its person (body), to judge how to contract out the property” (1988: 205). However, since prostitution is situated within patriarchal capitalism—and in the case of colonial Hong Kong within patriarchal capitalistic imperialism—which sometimes necessitates the selling of sexual services to assure women’s very survival, the “capacity to judge how to contract out the property (body)” is very much constrained. In other words, the seemingly private contract between the sex worker and her client forms part of a capitalistic enterprise liable to exploitation.

That colonial administrators were also aware of this is apparent in the way discussions around the question of the Chinese prostitute as a free agent arose and influenced the discourse around CD ordinances putting a check on Chinese brothel slavery. Governor Weld of the Strait Settlements, a colony with similar demographic, climatic, and cultural characteristics to Hong Kong (Levine 1998: 675 f.), wrote in one of his letters:

“The Protector of Chinese does what he can … [but] without the doctors he is helpless … he cannot change the relations which exist between the mistresses and the girls under a system where girls are brought up in China to be prostitutes, and are then bought by their mistresses, who bring them here. Here by law they are free, but they have no where to go; they have no money till they have earned it in the brothels; they are dependent on the brothel keepers for food and clothing, and, in fact, call her ‘Mother’ … in fact, any one going into one of these places … would … find nothing in their appearance or manner to indicate that they are prostitutes, nor would he see any signs of discontent or unhappiness, so readily do the Chinese accept any custom or position which is in accordance with traditionary usage.”^35^

Similarly centering the role played by brothel managers within Hong Kong’s colonial sex trade, Andrew and Bushnell point out that this capitalistic exploitation of sex workers was also practiced by Chinese women. For those with the monetary means to commence such an enterprise, becoming a brothel manager was a lucrative option for generating an income for themselves and their families.^36^ This capital, however, was often provided through the women’s affiliation with white men, gaining the status of “protected women” as a kind of exclusive, mistress-like relationship and obtaining privileges such as a paid residence or gifts.^37^ According to Andrew and Bushnell,

“These ‘protected women,’ enriched beyond anything they had even known before the foreigner came to that part of the world … could think of nothing so profitable as securing women and girls to meet the demands of the foreigners … it was but one step from that attitude of mind to the selling of girls to the foreigner, and the rearing of them for the object” (Andrew & Bushnell 1907: 19).

Dr. Ernst Johann Eitel, a sinologist and protégé of Pope-Hennessy, further added that

“almost every ‘protected woman’ keeps a nursery of purchased children or a few servant girls who are being reared with a view to their eventual disposal, according to their personal qualifications, either among foreigners here as kept women, or among Chinese residents as their concubines, or to be sold for export to Singapore, San Francisco, or Australia” (ibid.).

This illustrates how, in response to colonial demands, local agents became entwined with the patriarchal imperialistic capitalism that characterized the sex trade in colonial Hong Kong.

Freedom, however, is not only significant in considering the offering of sexual service but very much integral within the discourse of regulation under the CD ordinances. An adamant opponent of domestic CD acts, John Stuart Mill was labeled “a feminine philosopher” by Vanity Fair in 1873 and is considered a feminist philosopher in more recent scholarship for his belief of women as men’s equals.^38^ Despite his famous “harm principle” justifying limitations on the personal freedom of a few for the benefit of the majority, he argued that CD ordinances denied “the security of personal liberty … almost entirely from a particular class of women intentionally, but incidentally and unintentionally … from all women” (Mill in Robson 1991b: 351). Mill pointedly called out the problematic nature of CD legislation, in effect and practice, that allegedly aimed to protect public health. However, according to Jim Jose and Casey McLoughlin, “Mill’s views turn out to be both geographically and culturally specific” (Jose & McLoughlin 2016: 278), limiting his opposition of CD ordinances to what he calls “civilized communities,” for this right to liberty did not apply to “backward states of societies in which the race itself may still be in it nonage” (Mill in Robson 1991a: 224). Thus, Mill’s opposition was also problematic: his critique of CD ordinances for protecting men’s access to sexual services while penalizing women for providing them—thus enhancing men’s liberty while restricting women’s—should have been applicable to all geographic positions (Jose, McLoughlin 2016: 257, 278 f.; Knüfer 2023: 53–79).

Nevertheless, Mill’s selective opposition highlights an important argument within the discourse of repeal: whether and in what way liberty existed for Chinese women and colonized women in general. As Jack Jin Gary Lee puts it, “Chinese women [were understood] as not only different due to their perceived traditions but also inferior persons whose freedoms could not exist without the colonial state’s intervention” (Lee 2023: 21), so that “Chinese women, as well as other women involved in the colonial sex trade, were made voiceless while their conduct took on meanings that hinged on the differing perspectives of male officials at Whitehall and the colony” (Lee 2023: 22).

Although the CD ordinances relied on “the fiction that prostitutes could be clearly and unambiguously identified” (Howell 2000: 336), the construction and subsequent performance of prostitution was a complex interplay between the medico-moral discourse around female sexuality as deviant and prostitution as dangerous, colonial epistemology’s intersection of race and sexuality and the resulting anxieties of interracial relationships and miscegenation, and expectations of the correct performance of white heteronormative masculinity. Additionally, the construct of the prostitute was informed by patriarchal imperialistic capitalism and situated within a discourse in which she was silenced, declared simultaneously unfree and unprepared for the bestowment of Western liberty, and, most importantly, regarded as a victim of the customs of a lesser culture.

Policing prostitution was then the only way that these “grown-up children … [who] cannot be expected to take the least care of themselves or to have any compunction at spreading disease” could be delivered from slavery as well as prevented from damaging the British population through the transmission of disease.^39^

## Regulating Prostitution in the Name of Humanitarianism and Public Health

The colonial CD ordinances in Hong Kong were principally framed as a gesture of humanity:

“The Contagious Disease Acts in England have for their object the protection of Soldiers and Sailors. The Contagious Diseases Ordinances here have not only that object, but have also for their object the protection of girls and women against brothel slavery and hence it is that the Colonial Government had to adopt the ‘registration’ and ‘licensing’ of brothels, and keep up a complete Police inspection.”^40^

Humanitarianism and the self-imposed civilizing mission of the colonizer justified both the creation of the CD ordinances and their retention. On the protection of Chinese women from the brutalities of sexual slavery practiced in their own culture, Lord Kimberly wrote, “*On the ground of humanity*, we cannot shrink from this duty” (Stansfeld 1882: 35). Levine describes this as “humanitarian protectionism alongside medical necessity” (2001: 163), apparent in the way that even after outlawing compulsory medical examinations, the registration and regulation of brothels continued to be legal in Hong Kong, justified by the need to protect sex workers from bad working conditions and human trafficking (ibid.).

Institutions such as the Po Leung Kuk (PLK) exemplified this approach. Established in 1878, the PLK was created by members of the Chinese middle and upper-class elite to address human trafficking and provide shelter to women and children deemed victims of abuse or kidnapping. However, its activities extended beyond charity, functioning as a mechanism of social control. Women brought to the PLK were categorized based on their perceived moral and social value. Those deemed “victims” received training in domestic skills and were often reintegrated into society through marriage or employment. In contrast, women labeled “deviant”—such as prostitutes or those with contagious diseases—were segregated or deported, reinforcing colonial ideals of moral and racial hygiene. The PLK thus blurred the line between humanitarianism and discipline, reflecting the broader colonial project of regulating marginalized populations under the guise of benevolence (Chin 2012: 41; Chin 2013).

This adamant focus on the humanitarianism of colonial CD ordinances was closely linked to the framing of regulated prostitution as inherently Chinese and thus something colonial authorities had to accept in accordance with their promise to uphold Chinese laws and customs in Hong Kong. Andrew and Bushnell, however, criticized this logic:

“It was claimed that this slavery, and also domestic slavery, which sprang up so suddenly after the settlement of Hong Kong by the British, was the outgrowth of Chinese customs, and could not be suppressed but with the greatest difficulty, and their suppression was an unwarrantable interference with Chinese customs, Sir Charles Elliott having given promise from the first that such customs should not be interfered with. But, as we have shown, that promise was only made, ‘pending Her Majesty’s pleasure,’ which had been very plainly and pointedly expressed later as opposed to slavery” (Andrew & Bushnell 1907: 21).

Another interpretation Levine puts forth in one of her articles is the inherent Britishness of the CD legislation, with the laws representing enlightened— therefore British—fairness and humanity in contrast to Asian brutality and injustice, with an emphasis on protecting the individual (for example, the soldier in danger of being infected or the Chinese sex worker exploited by her pimp) as opposed to alleged Asian indifference to individual fate, given their traditions and customs. The homogenization of Chinese opinion regarding the practice of prostitution is once again apparent within this discourse, as well as the repeated highlighting of a necessary difference between the British and colonial Chinese legal subject, which adamant CD act critics like John Stuart Mill were unfortunately guilty of (Levine 1998: 686 f.; Jose & Mcloughlin 2016: 274).

While proponents of the CD ordinances argued that they played a key role in reducing the Hong Kong sex trade through compulsory brothel and sex worker registration, the legislation penalizing the trafficking of women and girls and the CD ordinances presented two separate legal tracks.^41^ Not until the 1880s, with the repeal of compulsory medical examinations but the continuation of brothel registration, was the trafficking of women penalized by the Hong Kong Protection of Women and Girls Ordinance of 1889.^42^ As Levine writes, “if the local CD ordinances had been framed, as was so often argued, primarily to procure and protect the apparently fragile liberty of Chinese women, it would surely have made sense, and saved money, to have combined the related laws far earlier” (1998: 694). Nevertheless, the new ordinance received very little attention and was seldomly invoked, marking a stark contrast to the rigid enforcement of the disease-focused ordinances before and contradicting the argument that CD ordinances were a humanitarian enterprise.^43^

Despite their apparent failure as a humanitarian measure, the ordinances were often cited as protecting against disease and reducing slavery. Even after the repeal of the CD ordinance in Hong Kong, the Lancet wrote in 1890:

“by enforcing rigorous examination by an honest and capable medical man, a check is immediately given to a form of slavery vicious in its idea and practice, but still more vile in that it is the chief cause whereby venereal disease is spread. Still more markedly do the above remarks apply to Chinese women in brothels, who are to a great extent absolute slaves to their mistress even in this colony under British rule. The abolition of compulsory examination withdrew the last chance of a certain degree of protection from their being compelled to practise prostitution whilst suffering from disease.”^44^

This opinion was shared by Colonial Surgeon Dr. Murray, who additionally refuted accusations that such examinations were disdained by the indigenous population:

“All known prostitutes, with the exception of a few Europeans and some half-caste Portuguese who ought not to have been exempted, were examined on an average once a week and if found diseased were detained in hospital; and very few, once I had got the system in proper working order, made any objection, soon being satisfied that it was for their advantage to be so dealt by.”^99^

However, such claims to humanitarian and medical benefit for both the indigenous and settler populations were disproved not only by the legal difference between Chinese sex workers who catered to Chinese clients versus US-European ones, and their respective requirements for medical examination, but by the lack of colonial healthcare available to the indigenous population. Colonial healthcare centered on the British military with preventative and protective measures to guarantee its strength and health and therefore the functioning of the colonial enterprise. The Chinese population in Hong Kong lacked both access to Western healthcare and adequate sanitary measures, with lock hospitals the only medical institutions providing well-funded medical treatment for Chinese residents, confirming the primary function of colonial healthcare as solely beneficial for the colonizer (Levine 2001: 165 f.).

Nevertheless, the prevention and protection of the military from VD proved unsuccessful despite claims to the contrary. On March 12, 1870, Dr. Maccoun wrote to Dr. Murray:

“Since my arrival on this station in the spring of 1868 I have been struck with the benefit derived from the system of registration and inspection of public women so ably carried out in this colony … During our visits to Hong Kong but few have been infected, and the type of the disease is mild … Out of a crew of 470 men, only five cases of chancre have been noticed.” 98

In another letter to Dr. Murry, Dr. W. Home, Principal Medical Officer of the Army, wrote:

“On behalf of the army medical officers stationed here, I have pleasure in expressing to you the great advantage we have received from the excellent sanitary arrangements in force in regard to prostitution … Venereal disease, from being but a few years ago one of the most common causes of unfitness for duty here has now become of comparatively rare occurrence” (ibid.).

This praise for the efficiency of the CD ordinances is summed up in yet another letter to Dr. Murray from Inspector General Robert Pottinger, stating that “Judging from my own experience I should say that syphilis has *all but* disappeared from the colony” (ibid.). This, however, does not add up with an investigation conducted by Andrew and Bushnell, based on the unpublished statements and statistics regarding unfitness for duty during that same period and findings of Pope-Hennessy’s Inquiry:

“In 1867 (after ten years’ experience) the public was informed that the Ordinance had been ‘on trial for nearly ten years, and had done singular service’ … Yet in this very same year – 1867, April 19th – Dr. Murray stated in an Official Report not intended for publication, but found by the Commission among other Government papers, and published, – ‘That venereal disease has been on the increase, in spite of all that has been done to check it, is no new discovery; it has already been brought before the notice of His Excellency.’”

“This same Dr. Murray’s Annual Report for the Public for 1867, was actually put in evidence before the House of Lords’ committee on venereal diseases … ‘Venereal disease here has now become of comparatively rare occurrence.’ Yet the Army Report for the previous year … states that ‘the admissions to hospital for venereal disease were 281 per 1000 men;’ i.e., more than one man in four of the whole soldiery had been in hospital for this ‘comparatively rare’ disease” (Andrew & Bushnell 1907: 32 f.).

One explanation for this failure was Hong Kong’s vicinity to China: “opposite Hong Kong is the main land of China, quite out of our control, and those people passed backwards and forwards without let or hindrance. If the whole country, as Malta, were under English Government, I think that registration and passport system, conjoined with the other regulations, would be effectual.”^45^ This rationale not only instrumentalizes Malta as the prime example of a successful CD ordinance in a colonial setting, but puts the blame on China, with its backward and brutal traditions imported into the colony by the large amount of Chinese immigrants, and its lack of modernity in the form of “registration and passport system”—two aspects crucially linked to Britain’s colonial project and internationalism—precluding the health and humanitarian benefits of the CD ordinances.

Thus the question arising from outcomes that contradicted the main legitimation of the CD ordinances is: What was the actual objective of the CD ordinances in Hong Kong? According to Governor and Pope-Hennessy, “The real purpose of the brothel legislation here has been, in the odious words so often used, the provision of clean Chinese women for the use of the British soldiers and sailors of the Royal Navy in this Colony” (Andrew & Bushnell 1907: 34), confirming the argument that colonial prostitution was necessitated by the colonizers’ need to display their dominance and superiority through the performance of white masculinity.

It should come as no surprise that not only was the sex act itself used as a form of power display, but the gynecological examinations that were part of the CD ordinances were utilized as a tool of dominance. In an inquiry into the alleged existence of Chinese slavery in Hong Kong, Pope-Hennessy’s commissioners found that the compulsory medical examination of Chinese sex workers serving a US-European clientele worsened their experience within the sex trade, with Governor Sir Richard MacDonnell describing compulsory examinations “as a species of penalty that may be inflicted whenever the expediency of such a measure was decided on.”^46^ Alfred Lister confirmed this before the Commission by stating, “new women would almost have preferred going to the whipping-post … the mere threat of sending them to examination was generally sufficient to keep them in order.”^47^

These tensions and negotiations between law and practice, rhetoric and intent were part and parcel of the colonial enterprise and “the delicacy required in ruling an alien civilization.”^48^ In his *History of Hong Kong*, Endacott finds that these “public health measures presented the policy of integrating the Chinese into the colonial administration with its greatest challenge” (1958: 183), namely the subjecting of only Chinese sex workers with US-European clients to regular medical examinations by CD legislation. As already mentioned, this particularity was informed by a theory of cultural and racial difference propagated by colonial medical and administrative authorities and situated within colonial epistemology and discourse as well as part of a larger project of disciplining and surveilling the Chinese population as a necessary measure for the security of the colonial project and the project of modernity (Levine 1998: 690; Howell 2004: 185).

## Regulationism as a Capitalist Enterprise

As considered in the second chapter of this paper, the capitalist nature of the colonial project cannot be excluded from an analysis of regulationism in colonial Hong Kong. The 288 complaints filed after the repeal of CD Ordinance 10 in Hong Kong in 1888,^49^ all of which were issued by clients claiming to have been infected with VD by a sex worker, seem to indicate that the purpose of the CD ordinances was indeed a reduction of infection. However, most of the complainants were individuals participating and profiting from the sex trade and the employment of sex workers,^50^ indicating that even after the appeal of its legislative embodiment, accusations of transmitting VD to clients still presented an instrument of discipline for those interested in keeping sex workers in line. This argument is supported by the stark disparity between the persecution and sentencing of sex workers and that of traffickers, brothel managers, and so forth.^51^

As the governor of Hong Kong, Pope-Hennessy, who was both part of the humanitarian network working toward the repeal of CD ordinances and acts in the British Empire and the abolishment of the colonial authority itself, was commonly characterized as liberal and “pro-native” (Lowe & McLaughlin 2008: 223–47). Having learned from his involvement in the anti-slavery movement in Barbados and the Windward Islands, where he also served as governor and sided with the Black population in their struggle against the islands’ white plantocracy, he seemed to have recognized patterns of slavery and dependency within the practice of imperial regulationism of Chinese prostitution in Hong Kong (Lambert & Howell 2003: 1–24). As Howell writes about Pope-Hennessys reaction to discovering the capitalist and exploitative nature of colonial regulationism,

“[he] was particularly exercised by the fact that one of the reasons that the system was introduced in the 1850s was to combat Chinese brothel slavery, and by the fact that, given that the government taxed brothels for Chinese clients, but did not examine their inmates, it was hard to avoid the conclusion that the government made money out of the trade. The British rulers of Hong Kong were little more than pimps and slave-masters” (Howell 2017: 190).

Pope-Hennessy thus benefited from his experience as an international agent in the Empire not only by recognizing patterns and particularities in legislation and practice but also by highlighting the contradictions originating from the tensions and negotiation within the colonial project, namely its facilitation of the continuation of slavery where it should have been antithetical to the alleged benevolence of the empire (ibid.).

By ordering the formation of a commission in 1877 to inquire into the workings of the CD ordinance in Hong Kong—following the deaths of A‑Sau and Tai-Yau in their attempt to escape arrest by the police—Governor Pope-Hennessy discovered both the ineffectiveness of the CD ordinances when it came to the eradication of VD and the reduction of so-called brothel slavery, with the latter, as mentioned before, regarded as separate from the treatment of VD. The inquiry found, for example, that Tai-Yau had sold her son into slavery to pay the $100 fine imposed on her by the inspector of brothels,^52^ highlighting the colonizer’s participation in a system they regarded as backward and inherently Chinese.^53^

The inquiry thereby revealed the lucrative benefits of the sex trade for the colonial enterprise, which is ultimately a capitalist endeavor. In the “Copy of Report of the Commissioners,” opponents of the CD ordinances calculated that the colony generated more than $100,000 in profit through the system of regulationism.^54^ In the end, the inquiry reached the conclusion that the CD ordinances were despotic, ineffective, and arbitrary in theory and practice.^55^

This conclusion, however, did little to sway the opinion of defenders and supporters of the CD ordinances. In their opinion, the alleged low morality of colonial legal subjects in Hong Kong and the fact that allegedly three quarters of Chinese women were engaged in the sex trade, meant that only unregistered sex workers and brothel managers would benefit from a repeal of the ordinance.^56^ Colonial Secretary Carnarvon, moreover, commented that “one cannot but remark the extreme tenderness shown by the Commissioners to the feelings and prejudices of the Chinese brothel-keepers and prostitutes,”^57^ highlighting the politics of gender all too present within the discourse. By portraying Pope-Hennessy as effeminate, he appears to confirm the colonial anxiety of the Orientalization and emasculation of white men in the colonies, who come to resemble the emasculated, weak Asian subject. His outrage and sympathies regarding the treatment of the local population were seen as an incorrect performance of white masculinity, whose purpose is to dominate and display power, not sympathize with those who are supposedly inferior to the prowess of white manhood.

Nevertheless, fellow critics similarly labored and investigated to highlight the capitalist exploitation connected to colonial regulationism in Hong Kong and elsewhere in the Empire. As James Stansfeld, for example, wrote, “the Government has reserved to itself the right to levy a duty on the bodies of women in Hong Kong, precisely as it has reserved to itself the right to levy duties upon brandy, tobacco and other profitable articles of consumption” (Stansfeld 1882: 4). He goes on to say:

“the arrest of the women enabled the officials to store those whose bodies were to be offered for sale, in the Government warehouses—the lawful, licensed brothels—and thereby to secure that the articles offered by them to the public should be periodically inspected and medicated by the surgeons hired by the Government for that purpose; it being held to be obvious that every male person whose custom it was to commit prostitution, would prefer that the bodies of the females purchased by him should be cleansed, licensed and warranted by the state, rather than by a private dealer” (ibid.).

Both are indicative of a colonial system of regulationism whose real purpose was neither the containment of contagion nor the protection of vulnerable individuals from prostitution but the calculated desire for fiscal profit, which was what ultimately defined the crown colony of Hong Kong.

## Shaping Prostitution, Shaping the Empire

The history of the British Empire is a tapestry woven with tensions, negotiations, and contestations. It gave rise to “discourses of difference,” constructing unstable, contingent categories shaped by assumptions about race, sexuality, gender, and class while simultaneously and relentlessly pursuing notions of superiority, modernity, and prestige. In this context, colonial prostitution was closely intertwined with the systems of patriarchy, imperialism, and capitalism that shaped, legitimized, and propelled the colonial enterprise, while being scapegoated for seemingly individual deviations and problems such as interracial sexual contact or lack of hygiene. Ironically, these issues were intrinsic to the very form of imperialistic capitalism that fueled the success of the British empire.

The CD ordinances, which may have presented themselves as medical measures based on humanitarian benevolence, were influenced by ideologies of racial, sexual, gender, and class inferiority and operated within systems of oppression, thus relying upon the intelligible performance of prostitution both for its theory and practice. The prostitute, however, was not a neatly defined, unambiguous category of risk, persecution, and culpability. Instead, she bore the mark of contemporary medico-moral discourse, embodying the construction of a racial and sexual other. Furthermore, her existence was firmly situated in the context of white masculine superiority and the imperialistic patriarchal capitalism of the period. The regulation of prostitution was fraught with contradictions. It failed to fulfill its lofty promises of public health protection and liberation from slavery, even after its repeal. Regulation, in essence, confirmed the capitalist enterprise that defined colonial prostitution at its core.

In conclusion, this study of the construction and regulation of colonial prostitution has highlighted the enduring continuities in colonial history: the complexities, contradictions, and challenges accompanying its every chapter, with the historian working tirelessly to comprehend the full extent of this multifaceted narrative. Further research into the performance and policing of prostitution is warranted, with a particular focus on the concept of slavery and the inter-empire understandings and negotiations regarding its form and definition as well as its subsequent connection to colonial prostitution. This research should also delve into the interconnectedness, similarities, and differences of colonial CD ordinances and their proponents and opponents, by utilizing the intersectional lens that is indispensable for conducting comprehensive, critical research.

## Footnotes

1 The term “prostitute,” as used in this paper, is critically employed with awareness of its historical and political implications. It reproduces the colonial language embedded in the medico-legal discourses of the period, which often pathologized and essentialized the identities of women engaged in sex work. By retaining this terminology, this study aims to maintain historical accuracy while simultaneously acknowledging its problematic nature and the need for a nuanced understanding of the experiences and agency of sex workers. For further discussions see Gilfoyle, T. J. (1999). Prostitutes in History: From Parables of Pornography to Metaphors of Modernity. *The American Historical Review* (104): 117–41.

2 Some scholars have sought to recover the voices of Chinese prostitutes in their research, making significant contributions to our understanding of their lives and identities. See, for example, Berney, Jane (2013). Writing Women’s Histories: women in the colonial record of nineteenth-century Hong Kong. *Women’s History Review* (22/2): 211–24.

3 For more information on the history of Hong Kong as a British colony, see Endacott, G. B. (1958). *A History of Hong Kong*. London: Oxford University Press, and Miners, Norman (1987). *Hong Kong Under Imperial Rule 1912–1941*. Hong Kong: Oxford University Press.

4 Correspondence Respecting the Alleged Existence of Chinese Slavery in Hong Kong. London 1882: 102.

5 “The conditions of life in Hong-Kong give to it a special stamp, one which must be borne in mind while considering the applicability to it of the Contagious Diseases Act … favour the presence and growth of prostitution.” The Contagious Diseases Acts in Hong-Kong. *The Lancet* (March 1, 1890): 480.

6 Alleged Existence of Chinese Slavery: 81.

7 No. 12 of 1857. An Ordinance for Checking the spread of Venereal Diseases. November 24, 1857. In *Historical Laws of Hong Kong*. URL: https://oelawhk.lib.hku.hk/items/show/118 (04.09.2023); “No. 10 of 1867. An Ordinance for the Better Prevention of Contagious Diseases” [July 23, 1867]. In: *Historical Laws of Hong Kong* (30.08.2023).

8 Seventeen out of forty-five British colonies enacted similar legislation: Contagious Diseases Ordinances (British Colonies). In: *Colonial Office for The House Commons* (London 1886): 2. Contagious Diseases Ordinances (British Colonies). In: *Colonial Office for The House of Commons* (London 1887): 3.

9 A Bill for the Prevention of Contagious Diseases at Certain Naval and Military Stations [June 20, 1864]. In: *U.K. Parliamentary Papers*. URL: https://parlipapers.proquest.com/parlipapers/docview/t70.d75.1864-039883?accountid=14571 (04.09.2023). For more information on Contagious Diseases Acts in Britain, see Siena, Kevin Patrick 2004. *Venereal Disease, Hospitals and the Urban Poor: London’s “Foul Wards,” 1600–1800*. Rochester: University of Rochester Press.

10 No. 10 of 1867.

11 In this way, the urban segregation of prostitution mirrors the all too apparent symbolism of East versus West.

12 Report of the Commissioners Appointed by his Excellency John Pope Hennessy CMG to enquire into the Workings of the Contagious Diseases Ordinance, 1867 (Hong Kong 1879): 28. For an important study on female brothel keepers in Hong Kong as entrepreneurs and agents, see Sinn, Elizabeth (2007). Women at Work: Chinese Brothel Keepers in Nineteenth-Century Hong Kong. *Journal of Women’s History* (19/2): 87–111.

13 For more information on how the CD ordinances were practiced in Britain and other British colonies, see Walkowitz, J. (1980). *Prostitution and Victorian Society: Women, class, and the state.* Cambridge: Cambridge University Press; Hyam, R. (1990). *Empire and Sexuality: The British Experience.* Manchester and New York: Manchester University Press; Ballhatchet, K. (1980). *Race, Sex, and Class Under the British Raj: Imperial Attitudes and Policies and their Critics, 1793–1905.* London: Weidenfeld and Nicholson; Warren, J. F. (1993). *Ah Ku and Karayuki-san: Prostitution in Singapore, 1870–1940.* Singapore: Oxford University Press; Van Heyningen, E. B. (1984). The Social Evil in the Cape Colony, 1868–1902: Prostitution and the Contagious Diseases Acts. *Journal of Southern African Studies *(10/2): 179–91.

14 Common justifications for this divergence within domestic and colonial legislation echo contemporary beliefs informed by colonial epistemology, for example: “It seems futile to attempt to legislate for Eastern races in these matters in the same way as for Europeans.” The Contagious Diseases Acts in Hong-Kong. *The Lancet* (March 1, 1890): 480.

15 No. 12 of 1857: 391.

16 See Butler, Judith (1990). *Gender Trouble: Feminism and the Subversion of Identity*. New York: Routledge; Butler, Judith (1993). *Bodies that Matter: On the Discursive Limits of “Sex.”* New York: Routledge.

17 Office War and Miscellaneous. *Colonial Office Papers*, CO 129/296 (4718), Public Record Office London.

18 See Crenshaw, Kimberlé (1991). Mapping the Margins: Intersectionality, Identity Politics, and Violence against Women of Color. *Stanford Law Review* (43/6): 1241–99.

19 In India, 20–25 percent of the hospitalization of military men in 1863 was a result of VD. See Royal Commission on the Sanitary State of the Army in India, Vol. I, Report of the Commissioners, London 1863, lxii. In *U.K. Parliamentary Papers*. URL: https://parlipapers.proquest.com/parlipapers/docview/t70.d75.1863-039148?accountid=14571 (04.09.2023). In 1868, The Lancet described VD in Hong Kong as “among the most common causes of unfitness for duty.” Contagious Diseases in China. *The Lancet* (November 14, 1868): 645.

20 See Said, Edward W. (1978). *Orientalism*. New York: Pantheon Books.

21 Annual Report of Colonial Surgeon 1874. In: Dispatches. *Colonial Office Papers*, CO 129/189 (13163), London 1880. Annual Report of the Colonial Surgeon 1879. In: Administration Reports. *Colonial Office Papers*, CO 131/11, London 1879–1884.

22 Report of the Commissioners: 5.

23 Copy of Report of the Commissioners to inquire into Working of Contagious Diseases Ordinances, 1867 (in Hong Kong) (London 1880): 20. In: U.K. Parliamentary Papers. URL: https://parlipapers.proquest.com/parlipapers/docview/t70.d75.1880-056310?accountid=14571 (04.09.2023).

24 Ibid.: 17.

25 Report of the Commissioners: 5. *Mui tsai* (little sister) describes the traditional Chinese practice of “adoption” in which young children, mostly girls, from poor families are sold off into domestic servitude. See also Leow, Rachel (2012). Age as a Category of Gender Analysis: Servant Girls, Modern Girls, and Gender in Southeast Asia. *The Journal of Asian Studies* (71): 975–90.

26 Proclamation. To the Chinese Inhabitants of Hong Kong (February 1, 1841). In: *Laws of Hong Kong. A Selection of Constitutional Documents, Conventions and Treaties. Appendix IV* (Hong Kong 1964).

27 See also MacPherson, Kerrie (2001). Health and Empire: Britain’s National Campaign to Combat Venereal Diseases in Shanghai, Hong Kong and Singapore. In: Roger Davidson and Lesley A. Hall (eds.): *Sex, Sin and Suffering. Venereal Disease and European Society since 1870*. London: Routledge: 175, 181; Howell. Prostitution and the Place of Empire: 186.

28 Copy of Report of the Commissioners: 17.

29 Report of the Commissioners: 56.

30 This exempts the few members of the Westernized Chinese elite of Hong Kong that were interviewed by Pope-Hennessy’s Commissioners. See Copy of Report of the Commissioners.

31 Report of a Departmental Committee on the Prevalence of Venereal Disease Among the British Troops in India (London 1897): 30.

32 This echoes the prominent argument employed in the discourse around legalization and regulation of sex work in early modern Europe, e.g. Venice, referencing male natural urges making sex work a necessity. Furthermore, as sex work was considered a “minor sin,” it could be employed as a preventative of “greater” sins such as incest or rape. See Clarke, Paula C. The Business of Prostitution in Early Renaissance Venice. *Renaissance Quarterly* (68): 420–23.

33 Report from the Royal Commission on the Administration and Operation of the Contagious Diseases Acts (London 1871): 17.

34 Copy of Report of the Commissioners: 19.

35 Correspondence relating to Repeal of Contagious Diseases Ordinances and Regulations in Crown Colonies (London 1889): 3.

36 The female-run business of prostitution has a long history both in early modern Europe and China, with Venetian sex workers often graduating to brothel managers after several years in the trade and Chinese sex workers buying and training young girls to become concubines, entertainers, and prostitutes, creating an interesting arena of female agency empowering women at the cost of exploiting other women. See Clarke, The Business of Prostitution (2015: 419–464); Cass, Victoria. (1999). *Dangerous Women: Warriors, Grannies and Geisha of the Ming*, Lanham, MD: Rowman & Littlefield Publishers; Hsieh, Bao Hua (2008). The Market in Concubines in Jiangnan During Ming-Qing China *Journal of Family History* (33): 262–90.

37 Copy of Report of the Commissioners: 18f.

38 Ward, Leslie. A Feminine Philosopher. *Vanity Fair* (March 29, 1873). See Jose, Jim and KCasey McLoughlin (2016). John Stuart Mill and the Contagious Diseases Acts. *Law and History Review*, (34): 249–79, and Mann, Hollie and Jeff Spinner-Halev (2010). John Stuart Mill’s Feminism: On Progress, the State, and the Path to Justice. *Polity* (42): 244–70.

39 Correspondence Repeal Contagious Diseases Ordinances: 22.

40 Registrar General of Hong Kong to Acting Colonial Secretary, 21 September 1882. In: Despatches. *Colonial Office Papers*, CO 129/203 (21149), London 1882.

41 No. 12 of 1857 and No. 10 of 1867 as the CD ordinances, No. 2 of 1875 as prohibiting the procuration and sale of women and children. See No. 2 of 1875. An Ordinance for the better Protection of Chinese Women and Female Children, and for the Repression of certain Abuses in relation to Chinese Emigration. In: Historical Laws of Hong Kong [March 18th, 1875]. URL: https://oelawhk.lib.hku.hk/items/show/338 (30.08.2023).

42 No. 19 of 1889. Protection of Women and Girls Ordinance. *Colonial Office Papers*, CO 129/243.

43 Only sixteen cases between January and May 1892 (Hong Kong Legislative Council, Proceedings of the Hong Kong Legislative Council, 1893, Hong Kong 1894, 625.).

44 The Contagious Diseases Acts in Hong-Kong. In *The Lancet* (March 1, 1890): 480.

99 (Murray 1897: 624).

98 (Murray 1897: 625).

45 Report of the Committee Appointed to Enquire into the Pathology and Treatment of the Venereal Disease, with the View to Diminish its Injurious Effects on Men of the Army and Navy (London 1868): 69.

46 Correspondence Respecting the Alleged Existence of Chinese Slavery in Hong Kong (London 1882): 91.

47 Ibid.

48 Note on the Legal and Practical Position of Brothels in Hong Kong, 16 May 1931. In: Position of Prostitution in Hong Kong, *Colonial Office Papers*, CO 129/533/10 (London 1931).

49 Hong Kong Legislative Council, Proceedings of the Hong Kong Legislative Council, 1889, Hong Kong 1890: 206.

50 Ibid.

51 Between 1859 and 1868, only 140 out of 411 persecutions were directed at brothel keepers. Copy of Report of the Commissioners: 24–27.

52 Copy of Report of the Commissioners: 6.

53 Ibid.: 2–6.

54 Ibid.: 62.

55 Ibid.: 63f.

56 Ibid.: 43.

57 Correspondence Relating to the Working of the Contagious Diseases Ordinances of the Colony of Hong Kong (London 1881): 25.
